# Pulmonary Sarcomatoid Carcinoma Associated with Arterial Thromboembolism in a Cat

**DOI:** 10.1155/2021/8849515

**Published:** 2021-01-13

**Authors:** Aria L. Guarino, Albert B. Jeon, Jeffrey R. Abbott, Richard C. Hill

**Affiliations:** College of Veterinary Medicine, University of Florida Small Animal Hospital, 2015 SW 16th Ave., Gainesville, FL, USA 32608

## Abstract

A 14-year-old, neutered male domestic shorthair cat presented for acute monoparesis with physical exam findings and biochemical data supportive of a distal arterial thromboembolism. Thoracic radiographs revealed an alveolar pattern in the right middle lung lobe and multifocal nodules in other lung lobes. A pulmonary mass was found on necropsy, which was composed of both carcinomatous and sarcomatous components, confirmed with cytokeratin and vimentin immunohistochemistry. Using the World Health Organization classification scheme for mixed pulmonary tumors, this tumor would be characterized as a pleomorphic squamous cell carcinoma under the umbrella term of pulmonary sarcomatoid carcinoma. The World Health Organization classification of mixed pulmonary tumors and its application to previously reported mixed pulmonary tumors in companion animals is discussed. This is the first reported case of this tumor type in a cat, as well as the first report of this tumor type associated with an arterial thromboembolism in any veterinary species.

## 1. Introduction

**Figure 1:**
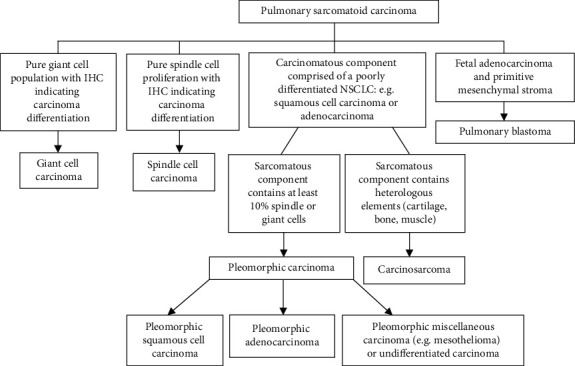
WHO classification of mixed pulmonary tumors. Pulmonary sarcomatoid carcinomas have five subtypes, the differentiating features of which are listed here. Note that the primary differentiating feature between “carcinosarcoma” and “pleomorphic carcinoma” is the presence of heterologous elements within the sarcomatous component of the carcinosarcoma. In human beings, sarcomatous components such as leiomyosarcoma, osteosarcoma, rhabdomyosarcoma, chondrosarcoma, liposarcoma, and angiosarcoma have been documented [3]. IHC: immunohistochemistry; NSCLC: non-small-cell lung carcinoma.

In human beings, pulmonary tumors consisting of epithelial and mesenchymal components are rare, aggressive tumors accounting for <1% of all lung tumors [1, 2]. The World Health Organization (WHO) uses the term “pulmonary sarcomatoid carcinoma” (PSC) as an umbrella term for mixed pulmonary tumors [1]. According to the WHO classification scheme, there are five subtypes of PSC: pleomorphic carcinomas, spindle cell carcinomas, giant cell carcinomas, carcinosarcomas, and pulmonary blastomas [1]. The differentiating features of each subtype are displayed in Figure 1.

Here, we report a cat with a PSC that presented for acute recurrent monoparesis suspected to be secondary to an arterial thromboembolism (ATE) based on biochemical data and clinical signs. The PSC is further classified as a “pleomorphic squamous cell carcinoma” using the WHO criteria [1].

## 2. Case Presentation

A 14-year-old, neutered male domestic shorthair cat was presented to the referring veterinarian for lameness with no history of trauma. The patient had a flaccid, cold right forelimb, poor appetite, and had lost 20% of his body weight over the previous 2 months. He was treated with an unspecified nonsteroidal anti-inflammatory drug and a glucosamine-chondroitin supplement. The lameness and poor appetite gradually resolved.

One month later, the patient was presented again for acute left hind limb lameness and vocalization. The distal left hind limb felt cold and was assessed to be painful. Conscious proprioception and the withdrawal reflex were absent, and femoral pulses were weak in that limb. Paw pads were pale compared to the contralateral limb. His heart rate was 140 beats per minute, and systolic blood pressure in an unspecified limb was 130 mmHg. An automated biochemistry analysis and complete blood count of a blood sample from an unspecified vessel showed increased blood concentrations of lactate (6.5 mmol/L; reference range 0.50-3.20), glucose (274 mg/dL; reference range 70-130), creatinine (2.0 mg/dL; reference range 0.8-1.8),and granulocytosis of 18,700 cells/*μ*L (3500-12,000). A urine specific gravity was 1.011.

On presentation to the author's hospital, the patient was tachycardic (200 beats per minute) and had a mildly increased respiratory rate (32 breaths per minute) and effort. The nail beds of the left hind limb were cyanotic. Femoral pulses were strong bilaterally, but no pulse was detected in the distal left hind limb either by palpation or with a Doppler flow detector. Plasma lactate and glucose concentrations were 13.4 mmol/L (reference range 0.4-1.5) and 105 mg/dL (reference range 87-111), respectively, in blood obtained from the lateral saphenous vein of the left hind limb; 7.2 mmol/L and 186 mg/dL, respectively, in blood obtained from the lateral saphenous vein of the right hind limb; and 2.1 mmol/L and 166 mg/dL, respectively, in blood obtained from the cephalic vein in the right forelimb. These findings suggested a compromise of blood flow in the left hind leg secondary to an ATE or tumor embolization.

**Figure 2:**
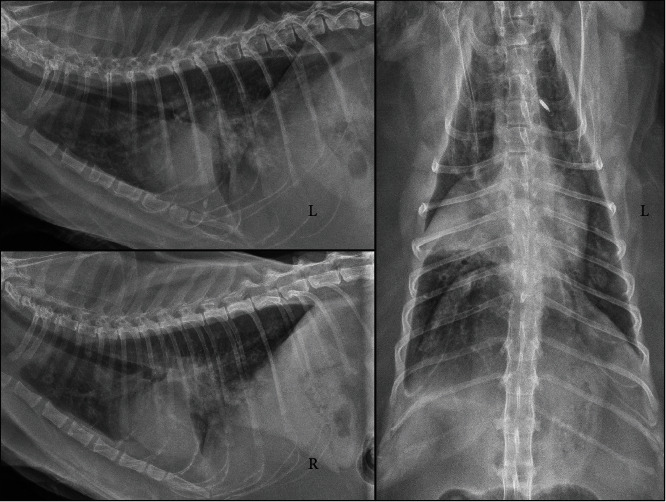
Thoracic radiographs. An alveolar pattern in the right middle lung lobe, diffuse pulmonary nodules, and mild pleural effusion are present.

On thoracic radiographs (Figure 2), an alveolar pattern was visible in the right middle lung lobe and multifocal nodules were visible in other lung lobes. An echocardiogram, abdominal and thoracic ultrasounds, and heartworm antibody and antigen testing did not reveal any additional abnormalities to explain the signs. Despite empirical treatment for arterial thromboembolism, the patient spontaneously arrested and the remains were submitted for necropsy. Owner consent was obtained to use the remains for research.

A large infiltrative mass effaced and expanded the parenchyma of approximately 80% of the right cranial lung lobe. Numerous, randomly distributed, tan, soft to mildly firm, pulmonary plaques and nodules, ranging from 2 × 0.8 × 0.8 cm to 0.1 × 0.1 cm × 0.1 cm, were embedded in the parenchyma and elevating the pleural surface throughout all lung lobes. The unaffected areas of the lung were dark red mottled, with small amounts of serosanguinous fluid on cut surfaces. The tracheobronchial lymph node was irregular, firm, and markedly enlarged, measuring 3.5 × 3.5 × 1.8 cm, with tan, homogenous cut surfaces. A grossly visible arterial thromboembolism was not identified.

**Figure 3:**
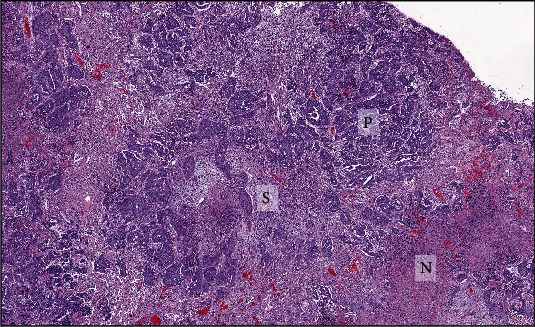
Subgross image. Hematoxylin and eosin staining showing trabeculae and nests of polygonal cells (P), streams and bundles of spindle cells (S), and areas of necrosis (N).

**Figure 4:**
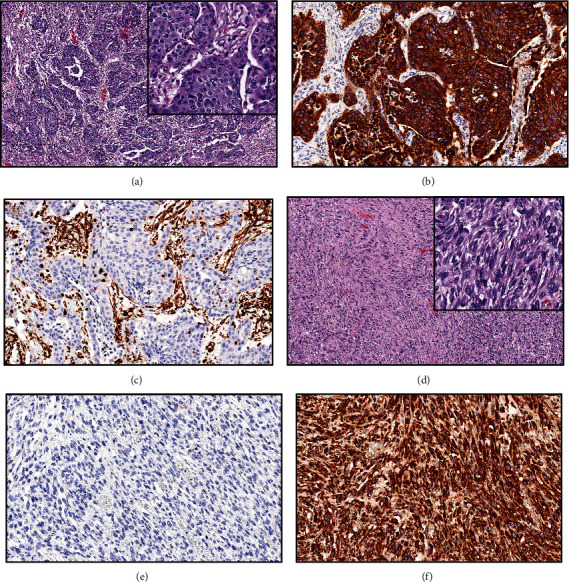
Histopathology. The polygonal cell population is represented in panels (a–c). Hematoxylin and eosin section with high magnification insert (a). Note the trabeculae and nests of polygonal cells and intervening stroma with a high number of mitotic figures (see inset). A1/A3 cytokeratin IHC stain (b). Note the strong staining of the polygonal cells consistent with epithelial cells and lack of stromal staining. Vimentin IHC stain (c). Note the lack of staining of the polygonal cells and sparse stromal staining. The spindle cell population is represented in panels (d–f). Hematoxylin and eosin section with high magnification insert (d). Note streams and bundles of spindled cells. A1/A3 cytokeratin IHC stain (e). Note the lack of staining of the spindle cells. Vimentin IHC stain (f). Note the strong staining of the spindle cells consistent with mesenchymal cells such as a fibroblast. IHC: immunohistochemistry.

Microscopic examination of the lung revealed fairly well-demarcated, unencapsulated, pulmonary nodules composed of two morphologically distinct neoplastic cell types (Figure 3). Approximately 20% of the mass was comprised of neoplastic polygonal cells forming small to large, irregular islands and nests supported by moderate amounts of fibrous stroma (Figure 4(a)). These polygonal cells had basophilic granular cytoplasm and marked anisocytosis and anisokaryosis. Fourteen mitoses were observed per ten 400x fields (2.37 mm^2^). The remainder of the neoplasm was comprised of spindloid and fusiform mesenchymal cells forming compactly arranged, haphazard, streams, bundles, and swirls (Figure 4(d)). The spindle cells had indistinct cellular borders, with moderate anisocytosis and anisokaryosis. Twenty-three mitoses were observed per ten 400x fields (2.37 mm^2^). In both portions of the mass, there are multifocal, nodular to regionally extensive areas of necrosis.

Almost all polygonal cells within the mass were immunopositive with cytokeratin consistent with epithelial cells (Figure 4(b)). Most of the cells within the mesenchymal population were immunopositive with vimentin, consistent with mesenchymal cells (Figure 4(f)). The tracheobronchial lymph node was completely effaced by similar neoplastic cells exhibiting morphology of both epithelial and mesenchymal differentiation.

## 3. Discussion

In human beings, PSC is an umbrella term for pulmonary carcinomas that contain pleomorphic or mesenchymal elements. These tumors account for <1% of all lung neoplasms in people and generally have a poor prognosis [1, 2]. The overall survival of stage-matched, surgically resected PSC is worse than other non-small-cell lung carcinomas [3].

The sarcomatous component of these tumors occurs secondary to the upregulation of a genetic program called the epithelial-to-mesenchymal transition (EMT), which is responsible for embryogenesis, cancer invasion, and metastasis [3, 4]. The upregulation of EMT occurs secondary to activation of genetic mutations that have been associated with resistance to chemotherapeutics and tyrosine kinase inhibitors, which is one reason why these tumors are difficult to treat [3]. Once the EMT is activated, cells from the carcinomatous component undergo metaplasia to become the sarcomatous component. Immunohistochemistry (IHC) is used to identify overexpression of molecules such as vimentin that are associated with the upregulation of the EMT [3]. IHC is also used to identify specific tumor markers and genetic abnormalities.

The WHO recommends IHC and accurate histopathological subtyping for pulmonary tumors [1]. Accurate subtyping is critical for several reasons: (1) specific drugs are only approved for specific histopathological subtypes, (2) some drugs are contraindicated for specific subtypes, and (3) specific subtypes trigger targeted molecular testing for genetic biomarkers that (a) have prognostic implications or (b) are the targets for specific immunotherapy as a part of a “personalized medicine” treatment approach [1, 3].

Currently, the WHO classification scheme is not widely applied to veterinary patients. However, beginning to do so could improve the ability of future studies to retrospectively evaluate specific subtypes for prognostic indicators, prevalence, risk factors, and possible therapeutic targets.

There have been two previous reports of “pulmonary carcinosarcomas” in companion animals: in one cat and one dog [5, 6]. The feline neoplasm was described as being composed of areas of bronchiolar adenocarcinoma, squamous metaplasia, multinucleated giant cells, and undifferentiated sarcoma [5]. There were no heterologous elements. Using the aforementioned WHO criteria, this previously described feline carcinosarcoma would be subtyped as a pleomorphic adenocarcinoma rather than a carcinosarcoma.

The canine neoplasm was described as containing areas of epithelial cells forming acinar and squamous patterns, sarcoma, giant cells, and islands of osteoid and cartilage metaplasia [6]. Using the WHO classification criteria, the canine carcinosarcoma would remain as named due to the presence of cartilage and osteoid within the tumor.

The neoplasm described in this report differed from the previously reported veterinary tumors in that the epithelial component consisted entirely of squamous cells, and there were no tubuloacinar formations or giant cells. Using the WHO classification criteria, the tumor in this case would be classified as a pleomorphic squamous cell carcinoma. The strong expression of vimentin in the sarcomatous component suggests the upregulation of the EMT, while the expression of cytokeratin in polygonal cells confirms the carcinomatous component [3, 4].

This case is also the first report of a feline ATE associated with a pulmonary tumor with mixed carcinomatous and sarcomatous components. Necropsy did not grossly identify the site of the ATE. It is possible that the clot dissolved either antemortem as a result of treatment with anticoagulants and fluids or postmortem prior to necropsy. Nevertheless, the lack of distal pulses and monoparesis of the left hind limb, combined with lower plasma glucose and higher plasma lactate concentration in the left than in the right hind limb, are highly suggestive of an obstruction of blood flow to the limb either by an thromboemoblism or tumor embolization [7].

In cats, ATEs have been most commonly associated with cardiac disease and hyperthyroidism, but have also been associated with neoplasia and administration of steroid or megestrol acetate [8–18]. ATEs may occur secondary to thrombus formation or, rarely, embolization of tumor. A review of the literature regarding feline arterial thromboembolisms listed by the National Library of Medicine revealed a total of 20 cats with ATE thought to be secondary to neoplasia [8, 11–18]. Of these 20 cats with neoplasia, 14 cats had pulmonary neoplasia identified on radiographs [8, 11–17]. Of the 14 cats with pulmonary masses, 9 of these were histopathologically confirmed to be pulmonary carcinoma [8, 12–15]. Four out of these 9 cats were found to have tumor cells within the emboli on histopathology [8, 12, 14].

In contrast to felines, human beings with pulmonary carcinomas more frequently acquire venous thromboembolism (VTE) than ATE [19]. Only a few instances of ATE as a result of spontaneous tumor embolization or tumor embolization at the time of surgery for pulmonary carcinoma have been reported [20–22]. In human beings with neoplasia, underlying causes of a hypercoagulable state include direct coagulation pathway activation, induction of inflammatory responses, inhibition of fibrinolytic activity, venous stasis, and vessel wall injury [23]. It has been estimated that VTE occurs in about 10% of ambulatory patients with lung neoplasia and the risk of VTE is twice as high for patients with pulmonary carcinoma than for patients with other pulmonary neoplasms [19]. Risk factors for VTE in human beings with pulmonary carcinoma include a cancer diagnosis of less than 6 months, hospitalization within 3 months prior to assessment, presence of cardiovascular risk factors, obesity, procoagulant phospholipid-dependent clotting time (Procoag-PPL) shorter than 44 seconds, and mean rate index of thrombin generation (MRI) lower than 125 nM/min [19]. We are unaware of any studies that have measured these coagulation parameters in cats. Future studies in cats could evaluate these specific coagulation parameters with the goal of identifying potential therapeutic targets for hypercoagulability.

## Data Availability

The clinical data used to support the findings of this study are included within the article and in a supplementary table.

## Abbreviations

ATE: Arterial thromboembolism
EMT: Epithelial-mesenchymal transition
IHC: Immunohistochemistry
NSCLC: Non-small-cell lung carcinoma
PSC: Pulmonary sarcomatoid carcinoma
VTE: Venous thromboembolism
WHO: World Health Organization.

## Supplementary Materials

Supplementary Materials Supplementary Table 1. Summary of previous literature describing cats with arterial thromboembolism secondary to neoplasia. References 8-18 were evaluated for cases of feline ATE secondary to neoplasia. References 8 and 11-18 included such cases and are included above.
