# Supplementary material for: Pulmonary Sarcomatoid Carcinoma Associated with Arterial Thromboembolism in a Cat
Source: Case Rep Vet Med. 2021 Jan 13;2021:8849515. doi: 10.1155/2021/8849515 (PMC7822706; doi:10.1155/2021/8849515)
Supplement: Supplementary Materials — Supplementary Table 1. Summary of previous literature describing cats with arterial thromboembolism secondary to neoplasia. References 8-18 were evaluated for cases of feline ATE secondary to neoplasia. References 8 and 11-18 included such cases and are included above. [file 8849515.f1.docx]

| **Reference** | **Cats with Neoplasia** | **Cats with Pulmonary Neoplasia** | **Cats with Histopathologically-Confirmed Pulmonary Carcinoma** | **Cats with Histopathologically-Confirmed Tumor Emboli** |
| --- | --- | --- | --- | --- |
| **[8]** | 6 | 2 | 2 | 1 |
| **[11]** | 3* | 3* | Not evaluated | Not evaluated |
| **[12]** | 1 | 1 | 1 | 1 |
| **[13]** | 1 | 1 | 1 | Not evaluated |
| **[14]** | 2 | 2 | 2 | 2 |
| **[15]** | 3 | 3 | 3 | Not evaluated |
| **[16]** | 1 | 1 | Not evaluated | Not evaluated |
| **[17]** | 1 | 1 | Not evaluated | Not evaluated |
| **[18]** | 2 | Not stated | Not evaluated | Not evaluated |
| **Total** | 20 | 14 | 9 | 4 |

**Supplementary Table 1.** Summary of previous literature describing cats with arterial thromboembolism secondary to neoplasia. References 8-18 were evaluated for cases of feline ATE secondary to neoplasia. References 8 and 11-18 included such cases and are included above.

*Two cases were included in analysis, and one case was presented in the discussion.
